# GTPase Pathways in Health and Diseases

**DOI:** 10.3390/cells11244055

**Published:** 2022-12-15

**Authors:** Yong Teng

**Affiliations:** 1 Department of Hematology and Medical Oncology, Winship Cancer Institute, School of Medicine, Emory University, Atlanta, GA 30322, USA; yong.teng@emory.edu; Tel.: +1-404-712-8514; 2 Wallace H. Coulter Department of Biomedical Engineering, Georgia Institute of Technology & Emory University, Atlanta, GA 30322, USA

GTPases, the molecular switches toggling between an inactive GDP-bound state and an active GTP-bound state, play a pivotal role in controlling complex cellular processes (e.g., cell differentiation, proliferation, and motility) and subcellular events (e.g., vesicle trafficking) by regulating a wide variety of signal transduction processes. Indeed, the dysfunction and deregulation of certain GTPases have been related to the development and progression of cancer and other disorders. Accumulating evidence demonstrates the therapeutic targeting of disease-specific GTPases as a promising way of saving patients. Therefore, it is worth exploring how GTPase signaling cooperates with other molecules and pathways during a defined biological or pathological response and how effective inhibitors to the activating potential for a given GTPase are developed.

In this Special Issue (SI), several contributors have provided mechanistic insights into GTPases and emphasized the ongoing strategies targeting their signaling cascades for future treatment. After a rigorous peer-review process, eight articles have been collected, consisting of two up-to-date reviews and six original research articles. Notably, these articles were contributed by renowned academic teams from North America, South America, Europe, and Asia, who are engaged in research regarding disease-associated GTPase signaling and regulators, demonstrating the great interest in this popular area of research. Our group contributed one review and one research article to inspire and motivate basic and clinical researchers to further solve puzzles within the world of GTPases.

Small GTPases are involved in many aspects of biology, including growth, differentiation, and development. Based on sequence and function, these monomeric GTPases have been grouped into five subfamilies: the Arf, Ras, Rho, Rab, and Ran subfamilies. The medical importance of small GTPases is highlighted by their amplification (e.g., Arf1) or mutational hyperactivation (e.g., Ras) in human cancers. Several approaches to the interference of small GTPases’ aberrant signaling, including the disruption of GTPase-GEF interaction, the enhancement of GAP activity, and the blockade of the activity of GTPase downstream effectors, have been explored and show promise in their ability to induce cancer remission. To stimulate efforts in this field, we systemically reviewed the functions of small GTPases and their modes of activation in cancer and discussed current challenges and perspectives regarding the development of anti-small-GTPase therapies [1]. Our research interests primarily lie in the determination of Arf1′s role in cancer biology and the development of effective counter strategies. Arf1 is a Ras-related small GTPase which plays a major role in vesicular trafficking. We and others have found that Arf1 is amplified in various tumors that drives cancer metastasis and progression [2,3,4]. Over the last few decades, several inhibitors with unrelated chemical structures have been discovered to inhibit the GEF-dependent activation of Arf1. Following our efforts to interfere with Arf1-GDP/Sec7-Arno, we explored the ability of constrained heteroaromatic γ-dipeptide scaffolds, so called 4-amino-(methyl)-1,3-thiazole-5-carboxylic acids (ATCs), to outline the key interacting elements of the Arno autoinhibitory domain. We rationally designed and synthesized constrained ATC-based γ-dipeptides to block Arf1 activation and examined their efficacy in head and neck squamous cell carcinoma [5]. Based on the observations in our previous study [6], we suggest that Arf1-targeting γ-dipeptides could be used in monotherapy or in combination with anti-EGFR agents for head and neck cancer treatment.

Cancer is characterized as a complex disease caused by coordinated alterations in multiple signaling pathways. The RAS/RAF/MEK/ERK (MAPK) signaling cascade, essential for cell inter- and intra-cellular crosstalk, is one of the best-defined pathways in cancer biology [1]. The overexpression and overactivation of members within the signaling cascade have been reported in many solid and blood cancers. Despite the enthusiasm surrounding this research area, how cancers will respond to drugs interfering with this pathway in the long term remains poorly understood. A review by Degirmenci et al. provides a comprehensive overview of the recent major findings in the study of this signaling cascade, particularly with respect to the impact on clinical cancer therapy [7]. This review also provides a conceptual framework for the development of more effective therapeutic approaches against RAS-driven cancers.

Vav1 works both as a catalytic RhoGTPase activator and an adaptor molecule, which is critical for T cell development and antigenic responses in a tyrosine-phosphorylation-dependent fashion. However, how Vav1 is modulated by the interacting partners and whether its biological activity is regulated by post-translational modifications are still unclear. Rodríguez-Fdez and his colleagues identified that Vav1 is positively modulated by phosphatidylinositol-5 phosphate (PI5P) and, possibly, other mono-PIs via direct protein–protein interactions [8]. Mechanistically, the affinity and specificity of Vav1-lipid interaction entail a new structural solution that involves the synergistic action of the Vav1 C1 domain and an adjacent polybasic tail. Using a collection of both acetylation- and deacetylation-mimicking mutants, the same group also revealed that the acetylation of four lysine residues on Vav1 downregulated the adaptor function of Vav1, inducing the stimulation of the nuclear factor of activated T cells [9]. Moreover, they found that additional acetylation sites were potentially involved in either the stimulation or downmodulation of specific Vav1-dependent downstream responses. It is worth noting that these studies did not exclude the possibility that other Vav family members could be regulated by lysine acetylation as well. Indeed, these new regulatory layers are not conserved in nonhematopoietic cells and Vav1 paralogs.

Another RhoGTPase is PLEKHG2, which can activate CDC42 by promoting the exchange of GDP for GTP on it. However, the role of PLEKHG2 in neurodevelopment remains to be determined. To address this question, a Japanese research team conducted in vitro and in vivo studies to explore the mouse Plekhg2 function during corticogenesis, including excitatory neuron migration, dendritic arbor formation, axon elongation, and spine formation [10]. One of the major findings was that PLEKHG2 plays an essential role in the maturation of axons, dendrites, and spines, and the blockade of its function could lead to neurodevelopmental disorders.

Uncontrolled inflammation processes are at the root of almost every major disease, including cancer, heart disease, diabetes, Alzheimer’s disease, and even depression. Few studies have involved the investigation of intracellular signaling pathways associated with the resolution of inflammation. In their study, Galvão et al. focused more on investigating the role of ROCK, a serine/threonine kinase, in a model of self-resolving neutrophilic inflammation [11]. They provided first-hand evidence that ROCK activity was correlated with the productive phase of acute inflammation and pharmacologically inhibiting it by Y-27632 decreased the accumulation of neutrophils in the inflammatory site by inducing apoptosis and neutrophil clearance. These findings highlight the importance of ROCK in the survival of neutrophil and the resolution of inflammation, suggesting that targeting ROCK could be developed into a compelling treatment to induce the resolution of established inflammatory responses.

Hypertension is a major risk factor for heart attack, stroke, and kidney failure. Anti-hypertensive therapies are commonly empirically prescribed and are often ineffective. GRAF3 is a Rho-specific GAP highly and selectively expressed in smooth muscle cells (SMC) in mice and humans, and it controls blood pressure by inhibiting RhoA-mediated SMC contractility. Using unique GRAF3-deficient mouse lines in combination with several well-characterized hypertensive models, a research team at the University of North Carolina reported that the modest ectopic expression of GRAF3 in SMC has the capacity to stably reduce blood pressure [12]. In this study, the authors also provided supporting evidence that endogenous GRAF3 cycling between inactive and active states was governed by post-translational modifications. These novel findings open avenues that targeting GRAF3 with pharmacological agents represents a new class of therapeutics to reduce the morbidity and mortality associated with hypertension.

The study of GTPases has sparked widespread enthusiasm for over 20 years. Tool compounds and lead drugs that pharmacologically inhibit GTPase pathways have shown promise. Recently, FDA granted accelerated approval to adagrasib for KRAS^G12C^-mutated non-small cell lung cancer. The articles within our SI add significant value to the development of mechanism-driven therapeutic strategies that highlight GTPases as promising targets. Thus, we expect these studies to have important implications for the improvement of translational medicine.
